# Interrogating population structure and its impact on association tests

**DOI:** 10.1186/1753-6561-5-S9-S25

**Published:** 2011-11-29

**Authors:** Huaizhen Qin, Robert C Elston, Xiaofeng Zhu

**Affiliations:** 1 Case Western Reserve University School of Medicine, Cleveland, OH 44106, USA

## Abstract

We found from our analysis of the Genetic Analysis Workshop 17 data that the population structure of the 697 unrelated individuals was an important confounding factor for association studies, even if it was not explicitly considered when simulating the phenotypes. We uncovered structures beyond the reported ethnicities and found ample evidence of phenotype–population structure associations. The first 10 principal components of the genotype data of the 697 individuals demonstrated much stronger associations with Q1, Q2, and the disease than did the individuals’ ethnicities. In addition, we observed that population structure was a confounding factor for the Q1-gene association when identifying the significant genes both with and without adjusting for the causal single-nucleotide polymorphisms, the ethnicities, and the principal components. Many false discoveries remained after adjusting for the causal single-nucleotide polymorphisms. Adjusting for the principal components appeared more effective than did adjusting for ethnicity in terms of preventing false discoveries. This analysis was performed with knowledge of the causal loci.

## Background

The 697 unrelated individuals in the Genetic Analysis Workshop 17 (GAW17) data set were from seven populations [1] (see the file unrelateds.ped). No population structure effect was directly incorporated into the simulation models to generate the three quantitative traits and the disease status. However, it was unclear whether population structure should be a concern for the analysis of this data set. Intuitively, the principal components (PCs) of the genotype scores and the reported individual ethnicities may capture different proportions of any overall population structure. We observed substantial additional structures within the populations by PC analyses of the population-specific and overall genotype data among the 697 individuals. We observed ample evidence for Q1–, Q2–, and disease–population structure associations by linear and logistic regression on the individual ethnicities and on the first 10 PCs of the genotype data of all 697 individuals. The PCs showed much stronger associations with the three phenotypes than did the ethnicities. We investigated confounding of the population structure on the Q1-gene association by contrasting the gene discoveries with and without adjusting for the 39 causal single-nucleotide polymorphisms (SNPs), ethnicities, and various numbers of PCs. Abundant false discoveries remained even after adjusting for the causal SNPs. In terms of preventing false discoveries, adjusting for the PCs appeared to be more effective than adjusting for ethnicity. In conclusion, it is necessary to adjust for population structure in association studies.

## Methods

### Interrogating hidden sample structures

The file unrelateds.ped [1] indicates that the 697 unrelated individuals in the GAW17 data set are from seven populations: Centre d'Etude du Polymorphisme Humain (CEPH)-, Denver Chinese, Han Chinese, Japanese, Luhya, Tuscans, and Yoruba (indexed by 1, …, 7, respectively, from now on). We performed population-specific and whole-sample PC analyses to uncover hidden population structures. For example, for the whole-sample PC analysis (PCA), let *G* = (*g_ij_*)*_n_*_×_*_M_* be the matrix of centered genotype scores (*n* = 697, *M* = 24,487); that is, *g*_1_*_j_* +⋯+*g_nj_* = 0 for each *j* ∈ {1, …, *M*}. We inspected the eigenvectors of *GG*′ to classify individuals.

### Uncovering phenotype–population structure associations

For individual *i*, let **t***_i_* = (*t_i_*_,1_, …, *t_i_*_,10_) be the first 10 PCs computed from *GG*′, and let **z***_i_* = (*z_i_*_,1_, …, *z_i_*_,6_) represent the 6 ethnicity contrasts defined by the seven populations (PS7); *z_i,p_* = 1 if *i* is from population *p* and 0 otherwise. Let Sex*_i_*, Age*_i_*, and Smoke*_i_* be standardized covariate scores, and let **x***_i_* = [1, Sex*_i_*, Age*_i_*, , Smoke*_i_*]. For each of Q1, Q2, and Q4, we tested **γ** = 0 under the model *y_i_* = **x***_i_***β** + **z***_i_***γ** + *ε_i_* and **δ** = 0 under the model *y_i_* = **x***_i_***β** + **t***_i_***δ** + *ε_i_* where *y_i_* is the trait value, *ε_i_* is random noise, **β** = (*β*_0_, …, *β*_4_)′, **γ** = (*γ*_1_, …, *γ*_6_)′, and **δ** = (*δ*_1_, …, *δ*_10_)′ are vectors of regression coefficients. For disease, we tested **γ** = 0 under the model logit[Pr(*y_i_* = 1)] = **x***_i_***β** + **z***_i_***γ** and **δ** = 0 under the model logit[Pr(*y_i_* = 1)] = **x***_i_***β** + **t***_i_***δ**. All the tests were conducted using the R functions lm(.), glm(.), and anova(.).

### Finding Q1-gene association

For individual *i*, let **s***_i_* = (*s_i_*_,1_, …, *s_i_*_,39_) and **g***_i_* = (*g_i_*_,1_, …, *g_im_*) be the vectors of genotypic scores of the 39 causal SNPs and a testing gene of *m* SNPs, and let *y_i_* be the trait value. We tested **η** = 0 under the linear regression models *y_i_* = **x***_i_***β** + **g***_i_***η** + *ε_i_*, *y_i_* = **x***_i_***β** + **s***_i_***θ** + **g***_i_***η** + *ε_i_*, *y_i_* = **x***_i_***β** + **z***_i_***γ** + **g***_i_***η** + *ε_i_*, and *y_i_* = **x***_i_***β** + **t***_i_***δ** + **g***_i_***η** + *ε_i_*, where **θ** = (*θ*_1_, …, *θ*_39_)′ and **η** = (*η*_1_, …, *η_m_*)′ are vectors of regression coefficients. We set **t***_i_* to be the first 10, 15, 100, and 200 PCs. All the tests were conducted using the R functions lm(.) and anova(.).

## Results

### Population structure

**Figure 1:**
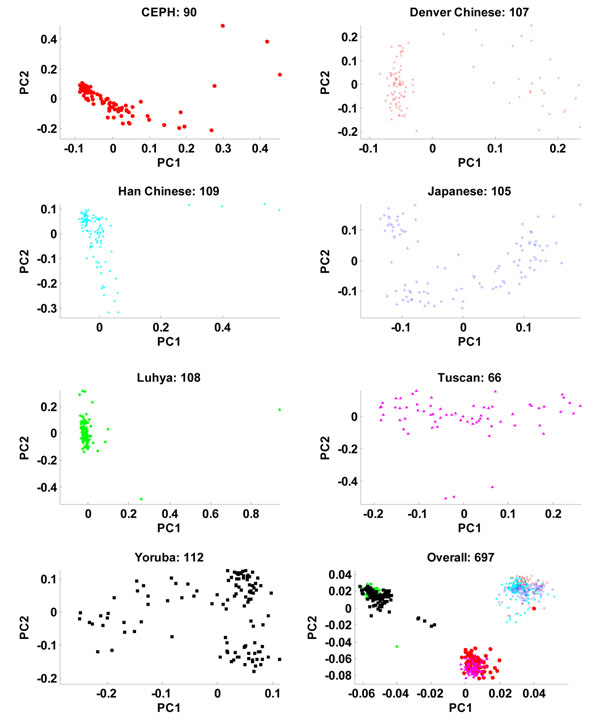
**Partial within- and overall-population structures.** Each of the seven populations has a specific within*-*population structure, as manifested by the first two principal components of the genotype matrix of the individuals in the population. Denver Chinese, Japanese, and Yoruba showed clear structures; Tuscan, CEPH, Han Chinese, and Luhya showed weak structures.

To better understand the population structure of the 697 individuals, we first performed a population-specific PCA (Figure 1). Each of the seven populations had a specific within-population structure, as manifested by the first two population-specific PCs. Denver Chinese, Japanese, and Yoruba showed clear structures; Tuscan, CEPH, Han Chinese, and Luhya showed weak structures. The PS7 vector would not be able to capture such subpopulation structures. The PCA of the genotypes of all 697 individuals uncovered additional structures.

### Phenotype–population structure associations

**Figure 2:**
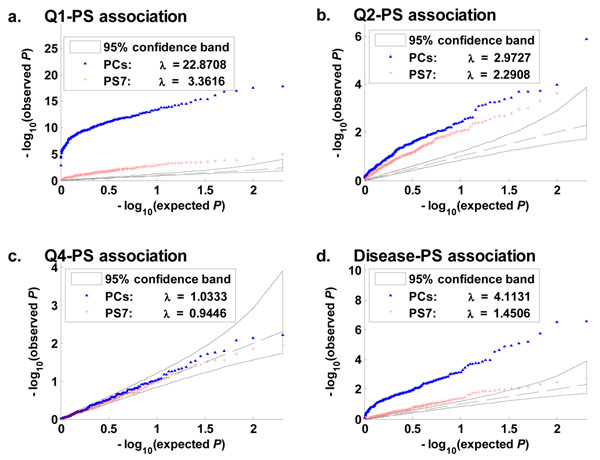
***Q-Q* plots of phenotype–population structure associations.** In each panel a–d, each point was computed from one of the 200 replicates. Phenotypes Q1, Q2, and the disease demonstrate clear associations with the individual ethnicities and demonstrate even stronger associations with the first 10 principal components. No clear evidence of Q4–population structure (PS) association was observed.

Phenotypes Q1, Q2, and the disease demonstrated clear associations with PS7 and demonstrated even stronger associations with the first 10 PCs (Figures 2a, b, d). For example, the *Q-Q* plot of the Q1-PS7 association was outside the 95% confidence band, and the genomic inflation factor of the 200 replicates was , where *P_j_* is the *p*-value of the test score for the *j*th replicate. The *Q-Q* plot of the Q1-PC association was even further away from the diagonal, with *λ* = 22.8708. Accordingly, the PCs better captured the population structure of the 697 individuals than did PS7. No clear evidence of Q4–population structure association was observed: The *Q-Q* plots of the Q4-PS7 and Q4-PC associations concentrated around the diagonal (Figure 2c). This result would be consistent with the fact that in the simulation Q4 was not influenced by any of the exonic SNPs.

### Q1-gene association

**Figure 3:**
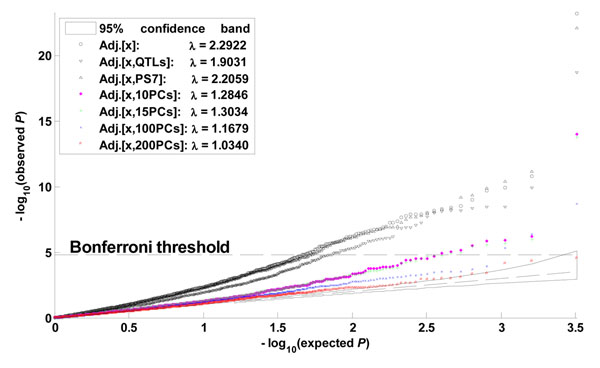
***Q-Q* plots of Q1-gene association.** This figure is based on the gene-specific *p*-values yielded by seven adjustments of environmental covariates, quantitative trait loci, ethnicity, and principal components when applied to replicate 10 of Q1. After Bonferroni correction, the seven adjustments identified 67 (2), 44 (2), 59 (2), 8 (2), 7 (2), 3 (2), and 0 (0) significant genes (causal genes), respectively.

The output of replicate 10 is presented in Figure 3. For each adjustment, we identified as significant those genes with *p*-values less than 0.05/3,205. In the simulated data for Q1, *FLT1* and *KDR* had the largest effects of all nine causal genes; *FLT1* consisted of 11 causal SNPs and 24 random SNPs, and *KDR* consisted of 10 causal SNPs and 6 random SNPs. We identified *FLT1* and *KDR* as the two most significant genes with all the adjustments discussed here except for that of 200 PCs. After adjusting for environmental covariates only, we identified 65 false discoveries, 42 of which remained even after adjusting for the 39 causal SNPs. This observation would explain the apparent Q1–population structure associations. After adjusting for ethnicity and environmental covariates, we identified 57 false discoveries. As anticipated, the number of false discoveries decreased as more PCs were used for adjusting. For example, we identified 8 (2), 7 (2), and 3 (2) significant genes (causal genes) after adjusting for the first 10, 15, and 100 PCs, respectively. However, the statistical power would be reduced if too many PCs were used for adjusting. For example, after adjusting for the first 200 PCs, we did not identify any genes as significant.

## Discussion

Using PCA of the 697 unrelated individuals in the GAW17 data set, we uncovered population structures in addition to their ethnicities and found ample evidence by linear and logistic regression analyses of phenotype–population structure associations and population structure confounding with phenotype-gene associations. The first 10 PCs of the genotype matrix of the 697 individuals showed much stronger associations with Q1, Q2, and the disease than did their ethnicities; and the PC adjustments appeared more effective than did the ethnicity adjustment in terms of preventing false discoveries. We still need to determine how to choose the optimal number of PCs, and what they are, for use in the adjustment.

We wondered whether the population structure was nonlinearly confounded with the phenotypes. Thus we also tested for phenotype associations with the first 10 PCs and ethnicities using least-squares kernel machines (LSKMs) [2,3], using linear, quadratic, Gaussian, and 2wayIX kernels (see [2-5] for details of LSKMs). All the results (not shown here) were similar to those in Figure 2. One remaining task is to find out why population structure has an effect here, because it was not explicitly put into the simulation models. Population history determines population structure, and population structure in turn affects the distribution of genotypes. We speculate that in the GAW17 data set the population history of many genes is similar to that of the true causal genes. This supposition could be verified by examining the canonical correlations between the PCs of the causal genes and the whole-sample PCs.

## Conclusions

Our analysis discovered that the population structure of the GAW17 unrelated individuals data is an important confounding factor, even though it was not explicitly involved as an independent predictor when simulating the phenotypes. It is thus necessary to adjust for any population structure, known or unknown, in association studies.

## Competing interests

The authors declare that there are no competing interests.

## Authors’ contributions

HQ conceived the project, analyzed the data and wrote the manuscript. RCE and XZ criticized and edited the manuscript. All authors read and approved the final manuscript.
